# Value of machine learning in predicting TAVI outcomes

**DOI:** 10.1007/s12471-019-1285-7

**Published:** 2019-05-20

**Authors:** R. R. Lopes, M. S. van Mourik, E. V. Schaft, L. A. Ramos, J. Baan Jr., J. Vendrik, B. A. J. M. de Mol, M. M. Vis, H. A. Marquering

**Affiliations:** 1 Department of Biomedical Engineering and Physics, Amsterdam UMC, University of Amsterdam, Amsterdam, The Netherlands; 2 Heart Centre, Amsterdam UMC, University of Amsterdam, Amsterdam Cardiovascular Sciences, Amsterdam, The Netherlands; 3 Technical Medicine, University of Twente, Enschede, The Netherlands; 4 Department of Clinical Epidemiology, Biostatistics and Bioinformatics, Amsterdam UMC, Amsterdam, The Netherlands; 5 Department of Radiology and Nuclear Medicine, Amsterdam UMC, University of Amsterdam, Amsterdam, The Netherlands

**Keywords:** Machine learning, Transcatheter aortic valve implantation, Outcome prediction, Prognosis

## Abstract

**Background:**

Transcatheter aortic valve implantation (TAVI) has become a commonly applied procedure for high-risk aortic valve stenosis patients. However, for some patients, this procedure does not result in the expected benefits. Previous studies indicated that it is difficult to predict the beneficial effects for specific patients. We aim to study the accuracy of various traditional machine learning (ML) algorithms in the prediction of TAVI outcomes.

**Methods and results:**

Clinical and laboratory data from 1,478 TAVI patients from a single centre were collected. The outcome measures were improvement of dyspnoea and mortality. Three experiments were performed using (1) screening data, (2) laboratory data, and (3) the combination of both. Five well-established ML techniques were implemented, and the models were evaluated based on the area under the curve (AUC). Random forest classifier achieved the highest AUC (0.70) for predicting mortality. Logistic regression had the highest AUC (0.56) in predicting improvement of dyspnoea.

**Conclusions:**

In our single-centre TAVI population, the tree-based models were slightly more accurate than others in predicting mortality. However, ML models performed poorly in predicting improvement of dyspnoea.

**Electronic supplementary material:**

The online version of this article (10.1007/s12471-019-1285-7) contains supplementary material, which is available to authorized users.

## What’s new?

- This is the first study on the prediction of TAVI outcomes using machine learning (ML) techniques.
- We have shown that ML techniques slightly outperform traditional methods in predicting mortality in our patient population; traditional logistic modelling has more prognostic value in predicting improvement of dyspnoea.
- N-terminal pro-b-type natriuretic peptide, body mass index, chronic kidney disease epidemiology collaboration, creatinine, and patient age were the most important features in our ML models.

## Introduction

Aortic valve stenosis (AS) is one of the most common valvular heart diseases, impacting, in general, the elderly population. In the past decade, transcatheter aortic valve implantation (TAVI) has developed into a routine treatment for AS patients at elevated risk of surgery. Although there is strict patient selection for the TAVI procedure and various planning and treatment support tools are available [1–3], a number of patients have limited benefit from TAVI [4]. Improved selection of these patients would allow increased benefit from the procedure and improve decision-making. Unfortunately, current risk models have only limited accuracy in predicting TAVI outcomes [5].

Previous clinical prediction models rely on traditional statistical regression models [6]. Alternatively, machine learning (ML), which is a computer science subdiscipline, has shown superior predictive value in various clinical areas, from detecting Alzheimer’s disease to identifying lung nodules [7, 8]. A more specific area of ML is supervised learning: with known outcomes, ML algorithms can learn automatically to optimise the prediction of this outcome. Moreover, ML techniques have outperformed conventional regression models when applied to a large amount of data [9].

Multiple risk models that have been used that are dedicated to the prediction of perioperative mortality and are not TAVI-specific, but intended for surgical aortic valve replacement such as the EuroSCORE, EuroSCORE II or the STS (Society of Thoracic Surgery) score [10, 11]. For TAVI, these are poor predictors of mortality and focus on procedural or 30-day mortality, as did the TAVI-specific TVT registry score [12]. The prediction of 1‑year mortality is even more challenging [13]. A more recent study also incorporated predefined features from computed tomography (CT) in combination with comorbidities to enhance the model [14].

We aimed to study the accuracy of various ML algorithms in predicting outcomes after a TAVI procedure. The accuracy was evaluated in the prediction of mortality and improvement of dyspnoea using a subset of well-established ML techniques.

## Methods

### Patient population

The database consists of 1,478 patients who underwent a TAVI between 2007 and 2018; their median age is 82.9 years [Q_1_ 78.0 − Q_3_ 86.4] and 55% of the patients are female. The data contain patient characteristics, medical history, symptoms, and test results prior to and after TAVI. Symptoms are dyspnoea, fatigue, collapse, and angina pectoris. Tests performed prior to TAVI are echocardiography, computed tomography angiography, coronary angiography, electrocardiography (ECG), and laboratory tests. Tests done after TAVI are echocardiography, ECG and laboratory tests.

**Fig. 1:**
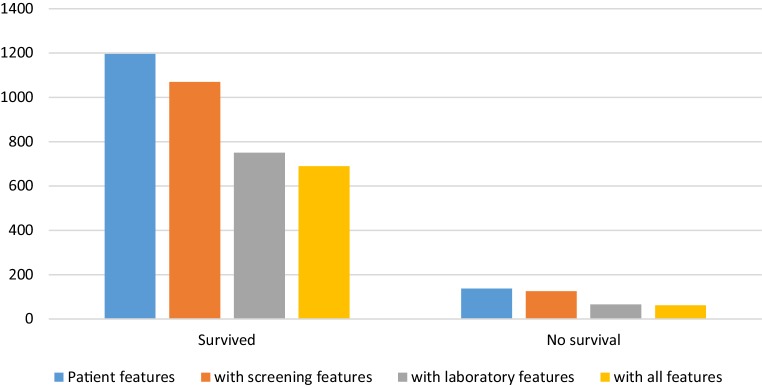
Number of patients without missing data per feature set for mortality outcome. For each feature set added, a lower number of samples is available due to missing values in different patients per set

**Fig. 2:**
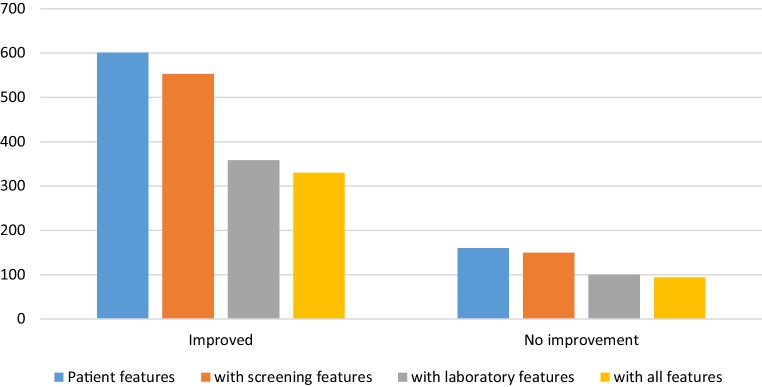
Number of patients without missing data per feature set for symptom outcome. For each feature set added, a lower number of samples is available due to missing values in different patients per set

The outcomes used are improvement of dyspnoea and mortality. Dyspnoea is measured using the New York Heart Association (NYHA) functional score (1–4). Mortality is defined as a patient who died of a cardiovascular disease within 1 year after the procedure. Patients with missing data are excluded. The baseline and 60-day follow-up NYHA score is known for 766 patients (605 improved, 161 non-improvements) and mortality is known for 1,400 patients (1,263 survivors, 137 non-survivors). For every outcome parameter, we performed three experiments: (1) using screening data; (2) using laboratory data; and (3) using both screening and laboratory data. The number of patients for each experiment is different due to missing values, as presented in Figs. 1 and 2. All variables, as well as the descriptive statistics, can be found in the Electronic Supplementary Material (Tables I and II).

### Clinical variables

The clinical variables used can be divided into three sets: patient characteristics, screening data, and laboratory data. The patient baseline characteristics included age, sex, body mass index (BMI), and access route chosen for the procedure. The screening data consist of the medical history, symptoms, and echocardiography prior to TAVI. The features used from this data are the existence of peripheral artery disease, chronic obstructive pulmonary disease (COPD), atrial fibrillation, diabetes mellitus (DM) in the medical history, left ventricular function, and aortic valve area assessed with echocardiography. The features used from the laboratory data are the pre-procedural values of N‑terminal pro-b-type natriuretic peptide (NT-proBNP), haemoglobin, albumin, chronic kidney disease epidemiology collaboration (CKD-EPI), and creatinine.

Based upon expert opinions we applied clipping to the NT-proBNP and creatinine variables, for values greater than 1,000 ng/l and 250 mmol/l, respectively. The nominal and categorical data were one-hot encoded; continuous features were normalised by removing the mean and scaling to unit variance, as requisite for many ML techniques [15]. Moreover, the COPD and DM were dichotomised to take into account the presence of the disease instead of the degree.

### Classification techniques

In this study, we selected a number of well-established ML techniques, which are: support vector machine (SVM) [16], random forest classifier (RFC) [17], multi-layer perceptron (MLP) [18], and gradient tree boosting (GTB) [19]. In addition, traditional logistic regression (LR) was also applied for comparison, since this technique is often used in clinical studies. All the implementations used in this project were provided by scikit-learn [15], except for GTB. We chose the XGBoost [19] library because of its GPU implementation, which speeds up training and optimisation.

To evaluate the models fairly, the database was split into two sets: a training and a testing dataset. The training data were used to find the optimal parameters for the classification task. The testing set was used to evaluate the trained model in unseen data, to ensure generalisation of the model and prevent the memorisation of the training set (overfitting). In this study, the models were evaluated with the Monte-Carlo cross-validation for 100 iterations and stratified splits of 70% for training and 30% for testing. With this large number of different training and testing sets, chances of having over-optimistic results are minimised. Moreover, to optimise the parameters of each model, a randomised grid search with stratified 5‑fold cross-validation was performed using the training set. The hyperparameters and ranges used for optimisation, including the weight penalisation applied to minimise the class unbalance issue, are available in the Electronic Supplementary Material (Tables III and IV).

Results of ML techniques are difficult to interpret. To elucidate which features may be important in the ML techniques, the average feature importance for RFC and GTB was calculated based on the number of times the feature was selected for splitting and weighted by the average squared improvement of the model over all trees [20].

### Performance assessment

The median of the area under the curve (AUC) of the receiver operating characteristic curve (ROC) from 100 iterations, using test sets, was selected to evaluate the performance of each model. To assess whether the difference in AUC between highest performing classifier and the other methods was statistically significant, the Wilcoxon signed-rank test was performed for each experiment. *p*-values < 0.05 were considered statistically significant.

## Results

The predictive value for improvement of dyspnoea was statistically significant but absent/low, with the best median AUC result of 0.56, using only laboratory features and LR. For mortality prediction, the model based on RFC was most accurate with an AUC of 0.70 [Q_1_ 0.67 − Q_3_ 0.74] and the results are considered to be significantly different according to the Wilcoxon test. All results are presented in Tab. 1.

**Table 1:** Median area under the curve [first and third quartiles] for all experiments. The *rows* are the machine learning technique and the *columns* are the set of features and the kind of outcome prediction. The highest-performing models and the models proved to be insignificantly different from those according to the Wilcoxon test are highlighted in *italics*

	Improvement of dyspnoea			1-year mortality		
Model	Screening	Laboratory	All	Screening	Laboratory	All
*GTB*	0.52[0.49–0.56]	0.53[0.50–0.55]	0.51[0.47–0.54]	0.65[0.62–0.67]	0.69[0.65–0.72]	0.69[0.66–0.72]
*SVM*	0.52[0.49–0.55]	0.52[0.48–0.56]	0.53[0.48–0.56]	*0.65[0.62–0.68]*	0.68[0.64–0.71]	0.69[0.65–0.72]
*MLP*	0.53[0.50–0.56]	0.52[0.48–0.55]	0.52[0.48–0.56]	*0.65[0.62–0.68]*	0.66[0.62–0.70]	0.66[0.62–0.71]
*RFC*	0.52[0.49–0.55]	0.53[0.49–0.56]	0.51[0.46–0.56]	*0.66[0.63–0.68]*	*0.70[0.67–0.73]*	*0.70[0.67–0.74]*
*LR*	*0.54[0.52–0.57]*	*0.56[0.52–0.58]*	*0.54[0.51–0.57]*	*0.66[0.63–0.69]*	0.67[0.62–0.70]	0.65[0.61–0.69]

*GTB* gradient tree boosting, *SVM* support vector machine, *MLP* multi-layer perceptron, *RFC* random forest classifier, *LR* logistic regression

**Fig. 3:**
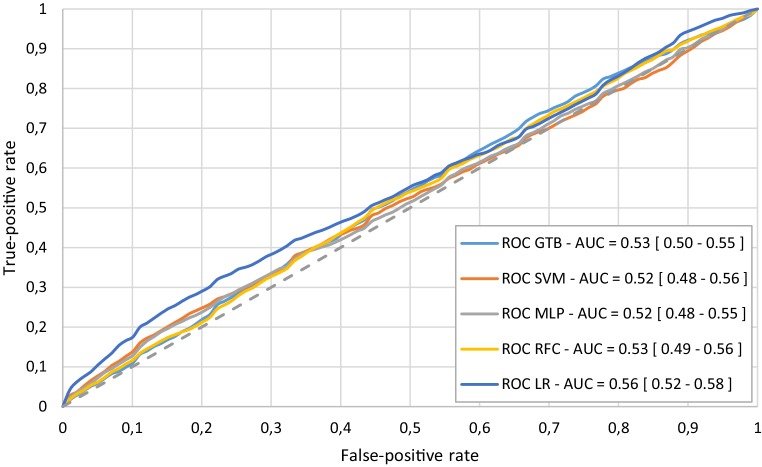
Median receiver operating characteristic (ROC) curve from 100 Monte Carlo cross-validation iterations for the prediction of dyspnoea improvement using laboratory features. *AUC* area under the curve, *GTB* gradient tree boosting, *LR* logistic regression, *MLP* multi-layer perceptron, *RFC* random forest classifier, *SVM* support vector machine

**Fig. 4:**
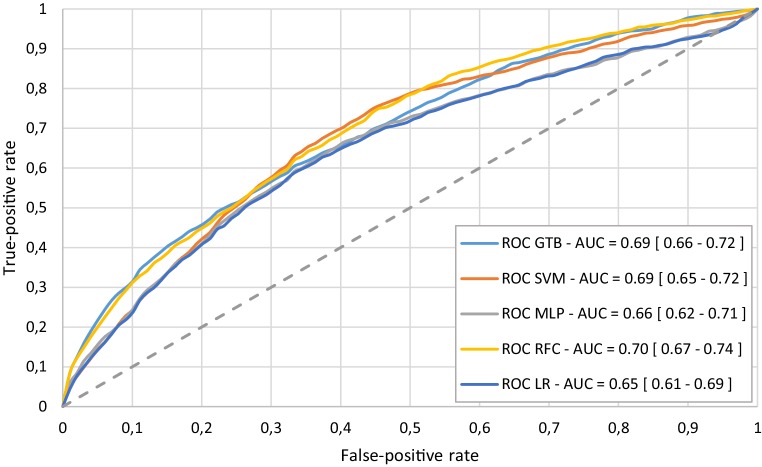
Median ROC curve from 100 Monte Carlo cross-validation iterations for the mortality prediction using all features. *AUC* area under the curve, *GTB* gradient tree boosting, *LR* logistic regression, *MLP* multi-layer perceptron, *RFC* random forest classifier, *SVM* support vector machine

The combination of the feature data sets did not result in an increased AUC in predicting improvement of dyspnoea. In mortality prediction, the models using the data combination showed similar AUCs using only laboratory features and all features. The median receiver operating characteristic (ROC) curves for the prediction of dyspnoea improvement (using the laboratory features) and mortality prediction (using all features) are displayed in Figs. 3 and 4, respectively.

The most relevant features for mortality prediction in GTB were determined by the importance of the features. In order of relevance, these were: NT-proBNP, BMI, CKD-EPI, creatinine, and age.

## Discussion

In our population of 1,478 patients who underwent a TAVI procedure, the selected subset of ML techniques had little added prognostic value in predicting mortality and improvement of dyspnoea compared to commonly applied LR techniques. In the prediction of mortality, ML techniques achieved similar scores using all features and only the laboratory features. The increase in prognostic value and the improvement of dyspnoea prediction was rather low, even with the combination of clinical and laboratory data.

Some recent studies that applied ML have often shown positive results for prognosis prediction. In the study of Memarian et al. [21], ML methods were applied to multimodal data (clinical data, electroencephalography, magnetic resonance imaging) to predict the outcome of surgery in patients with mesial temporal lobe epilepsy, achieving a prediction accuracy of 95% using SVM-derived classifiers. Frizzell et al. [22] compared ML methods to LR in predicting 30-day readmission in patients discharged following hospitalisation for heart failure. Similar to our findings these results did not show an improvement in prediction accuracy. The prediction of six cardiovascular outcomes (including heart failure and all-cause death) was assessed by Ambale-Venkatesh et al. [23], whereby random survival forests and other ML techniques were compared to the standard cardiovascular scores. They concluded that ML improved the accuracy of cardiovascular event prediction in initially asymptomatic patients.

Our results confirm that predicting outcomes of TAVI procedures is challenging. Many factors may impact the patient’s outcome, many of which are not considered in the modelling. The inclusion of more and different kinds of features, such as different examinations, CT scans, and ECG, is currently a subject of investigation. By including different sets of features and more complex models, the predictive value may increase.

There was no implicit order in the data variables that we tried to exploit. Also, no variables were transformed into a dense representation. We included all variables that were considered relevant by clinical experts. We are aware that one-hot encoding generates data sparsity. Even though one-hot encoding can downgrade the performance of some ML methods, it is an important step for distance-based methods such as the SVM. In our study, only a small number of categorical variables (with few classes) were one-hot encoded to prevent hampering the performance of methods due to data sparsity.

The methods used in this study are generalisable to other clinical challenges in which prediction of outcomes is warranted. It is expected that the application of ML techniques in combination with clinical knowledge will become increasingly important in coming years to improve prognostics. Models with higher accuracy may improve outcome prediction after TAVI, allowing a more individual approach in clinical care.

This study suffered from a number of limitations. The dataset used in this study may be one of the largest Dutch single-centre TAVI datasets available. However, with unbalanced measures (such as a relatively small population that did not survive the 1st year), the effect of the data is reduced. Moreover, many patients were excluded because of missing data, which can be mitigated by using imputation techniques. In this study, we chose symptom reduction using the NYHA classification and 1‑year mortality as outcome measures. Other outcome measures, however, might be relevant for the TAVI population.

## Conclusion

In our population of patients treated with TAVI, ML techniques were able to predict mortality using the current set of features. In predicting a reduction of dyspnoea, the traditional LR technique outperformed the others. Adding more features or increasing the dataset size may result in a situation in which ML techniques have more added value.

## Caption Electronic Supplementary Material

Summarized patient characteristics and hyperparameters used for optimisation.
