# Supplementary material for: Value of machine learning in predicting TAVI outcomes
Source: Neth Heart J. 2019 May 20;27(9):443–50. doi: 10.1007/s12471-019-1285-7 (PMC6712116; doi:10.1007/s12471-019-1285-7)
Supplement: Supplementary file 1 — Summarized patient characteristics and hyperparameters used for optimisation. [file 12471_2019_1285_MOESM1_ESM.docx]

Supplementary Table I – Summarized patient characteristics grouped by symptoms

|  |  | **Grouped by symptoms** | | |
| --- | --- | --- | --- | --- |
|  |  | **Missing** | **Improved** | **Didn't improve** |
| **variable** | **level** |  |  |  |
| **n** |  |  | 605 | 161 |
| **Gender** | **Female** | 36 | 334 (55.39) | 98 (60.87) |
|  | **Male** |  | 269 (44.61) | 63 (39.13) |
| **Body Mass Index** |  | 141 | 27.10 [24.50,30.40] | 26.40 [23.70,30.50] |
| **Age (years)** |  | 143 | 83.40 [77.90,86.60] | 82.70 [77.50,86.10] |
| **PAD** | **No** | 155 | 447 (74.13) | 112 (69.57) |
|  | **Yes** |  | 156 (25.87) | 49 (30.43) |
| **COPD** | **No** | 156 | 414 (68.66) | 114 (70.81) |
|  | **Yes** |  | 189 (31.34) | 47 (29.19) |
| **Atrial Fibrillation** | **No** | 171 | 350 (58.04) | 109 (67.7) |
|  | **Yes, unknown type** |  | 77 (12.77) | 14 (8.7) |
|  | **Yes, paroxysmal** |  | 84 (13.93) | 17 (10.56) |
|  | **Yes, permanent** |  | 78 (12.94) | 15 (9.32) |
|  | **Yes, persistent** |  | 14 (2.32) | 6 (3.73) |
| **Diabetes mellitus** | **No** | 166 | 417 (69.15) | 110 (68.32) |
|  | **Yes** |  | 186 (30.85) | 51 (31.68) |
| **Treatment for Diabetes** | **Diet alone** | 92 | 2 (0.33) |  |
|  | **Insulin** |  | 33 (5.46) | 5 (3.11) |
|  | **No** |  | 461 (76.32) | 129 (80.12) |
|  | **Oral medication** |  | 101 (16.72) | 23 (14.29) |
|  | **Oral medication and insulin** |  | 7 (1.16) | 4 (2.48) |
| **Left Ventricular Function** | **Good** | 147 | 371 (61.63) | 108 (67.08) |
|  | **Mildly impaired** |  | 110 (18.27) | 31 (19.25) |
|  | **Moderately impaired** |  | 74 (12.29) | 11 (6.83) |
|  | **Poor** |  | 39 (6.48) | 11 (6.83) |
|  | **Very poor** |  | 8 (1.33) |  |
| **Aortic Valve Area (cm^2^)** |  | 262 | 0.80 [0.70,1.00] | 0.81 [0.70,0.95] |
| **NT-proBNP (ng/L)** |  | 448 | 1644 [641,3957] | 1258 [595,3373] |
| **Hemoglobin (mmol/L)** |  | 170 | 7.79 (1.01) | 7.85 (1.05) |
| **Albumin (g/L)** |  | 453 | 42 [40,44] | 42.00 [39,45] |
| **CKD-EPI (ml/min/1.73 m^2^)** |  | 199 | 59.89 [46.03,75.27] | 59.00 [43.51,73.44] |
| **Creatinine (mmol/L)** |  | 174 | 88 [73,112] | 89 [71,113] |
| **Access Route** | **Direct aorta** | 162 | 72 (11.94) | 32 (20.0) |
|  | **Transapical** |  | 57 (9.45) | 21 (13.12) |
|  | **Transfemoral** |  | 474 (78.61) | 107 (66.88) |
|  | **Via arteria subclavia** |  |  | 0 (0.00) |

Supplementary Table II – Summarized patient characteristics grouped by 1-year mortality

|  |  | **Grouped by 1-year mortality** | | |
| --- | --- | --- | --- | --- |
|  |  | **Missing** | **Survived** | **Didn't survive** |
| **variable** | **level** |  |  |  |
| **n** |  |  | 1263 | 137 |
| **Gender** | **Female** | 36 | 696 (55.77) | 67 (48.91) |
|  | **Male** |  | 552 (44.23) | 70 (51.09) |
| **Body Mass Index** |  | 141 | 27.00 [24.20,30.40] | 25.50 [23.70,29.10] |
| **Age (years)** |  | 143 | 82.80 [77.80,86.20] | 84.00 [80.00,87.40] |
| **PAD** | **No** | 155 | 909 (75.19) | 84 (61.31) |
|  | **Yes** |  | 300 (24.81) | 53 (38.69) |
| **COPD** | **No** | 156 | 871 (72.16) | 74 (54.01) |
|  | **Yes** |  | 336 (27.84) | 63 (45.99) |
| **Atrial Fibrillation** | **No** | 171 | 734 (61.58) | 70 (51.09) |
|  | **Yes, unknown type** |  | 127 (10.65) | 28 (20.44) |
|  | **Yes, paroxysmal** |  | 161 (13.51) | 17 (12.41) |
|  | **Yes, permanent** |  | 137 (11.49) | 20 (14.6) |
|  | **Yes, persistent** |  | 33 (2.77) | 2 (1.46) |
| **Diabetes mellitus** | **No** | 166 | 833 (69.47) | 92 (67.15) |
|  | **Yes** |  | 366 (30.53) | 45 (32.85) |
| **Treatment for Diabetes** | **Diet alone** | 92 | 10 (0.8) |  |
|  | **Insulin** |  | 53 (4.24) | 7 (5.11) |
|  | **No** |  | 958 (76.7) | 104 (75.91) |
|  | **Oral medication** |  | 200 (16.01) | 23 (16.79) |
|  | **Oral medication and insulin** |  | 28 (2.24) | 3 (2.19) |
| **Left Ventricular Function** | **Good** | 147 | 756 (62.84) | 59 (43.38) |
|  | **Mildly impaired** |  | 227 (18.87) | 32 (23.53) |
|  | **Moderately impaired** |  | 138 (11.47) | 26 (19.12) |
|  | **Poor** |  | 71 (5.9) | 16 (11.76) |
|  | **Very poor** |  | 11 (0.91) | 3 (2.21) |
| **Aortic Valve Area (cm^2^)** |  | 262 | 0.80 [0.66,0.95] | 0.80 [0.66,0.97] |
| **NT-proBNP (ng/L)** |  | 448 | 1412 [569,3332] | 3365 [1541,7007] |
| **Hemoglobin (mmol/L)** |  | 170 | 7.80 (1.00) | 7.65 (1.17) |
| **Albumin (g/L)** |  | 453 | 42 [40,44] | 41 [38,43] |
| **CKD-EPI (ml/min/1.73 m^2^)** |  | 199 | 60.10 [45.90,74.54] | 48.27 [33.66,65.41] |
| **Creatinine (mmol/L)** |  | 174 | 88 [72,111] | 102 [78,154] |
| **Access Route** | **Direct aorta** | 162 | 214 (17.58) | 35 (25.55) |
|  | **Transapical** |  | 109 (8.96) | 23 (16.79) |
|  | **Transfemoral** |  | 894 (73.46) | 78 (56.93) |
|  | **Via arteria subclavia** |  |  | 1 (0.73) |
|  |  |  |  |  |

Supplementary Table III - Hyperparameters used for SVM

| **Classifier** | **Kernel Type** | **Penalty parameter *C*** | **Kernel coefficient γ** | **Degree of the Polynomial kernel** | **Class weight** |
| --- | --- | --- | --- | --- | --- |
| **SVM** | Linear | [0.001, 0.01, 0.1, 1, 10, 100] | n.a. | n.a. | [balanced, 1:5, 1:7, 1:10, 1:13, 1:15] |
|  | Radial basis function | [0.001, 0.01, 0.1, 1, 10, 100] | [1, 0.1, 0.01, 0.001, 0.0001] | n.a. | [balanced, 1:5, 1:7, 1:10, 1:13, 1:15] |
|  | Polynomial | [0.001, 0.01, 0.1, 1, 10, 100] | [1, 0.1, 0.01, 0.001, 0.0001] | [1, 2, 3, 4, 5, 6] | [balanced, 1:5, 1:7, 1:10, 1:13, 1:15] |
|  | Sigmoid | [0.001, 0.01, 0.1, 1, 10, 100] | [1, 0.1, 0.01, 0.001, 0.0001] | n.a. | [balanced, 1:5, 1:7, 1:10, 1:13, 1:15] |

Supplementary Table IV - Hyperparameters used for RFC, MLP and GTB

| **Classifier** | **Parameter Name** | **Parameter Value** |
| --- | --- | --- |
| **RFC** | Number of trees | [10, 20, 50, 100, 400, 800, 1200, 1600, 2000] |
|  | Max features for split | None, auto, sqrt, log2 |
|  | Quality of split | Gini, entropy |
|  | Max depth | [None, 3, 5, 7, 9, 11, 13, 20, 50] |
|  | Min samples per split | 2, 4, 6, 8, 10, 20 |
|  | Min samples per leaf | 2, 4, 6, 8, 10, 20 |
|  | Class weight | [balanced, 1:5, 1:7, 1:10, 1:13, 1:15] |
| **MLP** | Hidden Layer sizes | [4, 4], [4, 8, 4], [50, 25], [50, 25, 10], [70, 40, 20], [70, 30], [50, 30, 20, 10] |
|  | Regularization parameter | [0.1, 0.01, 0.001, 0.0001] |
|  | Batch size | [8, 16, 32, 64] |
|  | Learning rate | [0.01, 0.001] |
|  | Activations | [Relu, Logistic, TanH] |
|  | Optimization | Adam, L-BFGS |
| **GTB** | Minimum child weight | [1, 5, 10, 15] |
|  | Gamma | [0, 1, 3, 5] |
|  | Subsample ratio | [0.6, 0.8, 1.0] |
|  | Subsample ratio of columns | [0.6, 0.8, 1.0] |
|  | Max depth | [4, 7, 10, 15] |
|  | Class weight | [5, 7, 10, 13, 15] |
|  | Max delta step | [0, 1, 5, 10] |
|  | Number of trees | [30, 50, 100, 200] |
